# MiR-34c represses muscle development by forming a regulatory loop with *Notch1*

**DOI:** 10.1038/s41598-017-09688-y

**Published:** 2017-08-24

**Authors:** Lianjie Hou, Jian Xu, Huaqin Li, Jinxin Ou, Yiren Jiao, Chingyuan Hu, Chong Wang

**Affiliations:** 1 National Engineering Research Center for Breeding Swine Industry, Guangdong Provincial Key Lab of Agro-Animal Genomics and Molecular Breeding, College of Animal Science, South China Agricultural University, Guangzhou, Guangdong 510642 PR China; 2 Department of Human Nutrition, Food and Animal Sciences, College of Tropical Agriculture and Human Resources, University of Hawaii at Manoa, 1955 East-West Road, AgSci. 415J, Honolulu, HI 96822 USA

## Abstract

Since pork accounts for about 40% of global meat consumption, the pig is an important economic animal for meat production. Pig is also a useful medical model for humans due to its similarity in size and physiology. Understanding the mechanism of muscle development has great implication for animal breeding and human health. Previous studies showed porcine muscle satellite cells (PSCs) are important for postnatal skeletal muscle growth, and *Notch1* signaling pathway and miRNAs regulate the skeletal muscle development. *Notch1* signal pathway regulates the transcription of certain types of miRNAs which further affects target gene expression. However, the specific relationship between *Notch1* and miRNAs during muscle development has not been established. We found miR-34c is decreased in PSCs overexpressed *N1ICD*. Through the overexpression and inhibition of mi-34c, we demonstrated that miR-34c inhibits PSCs proliferation and promotes PSCs differentiation. Using dual-luciferase reporter assay and Chromatin immunoprecipitation, we demonstrate there is a reciprocal regulatory loop between *Notch1* and miR-34c. Furthermore, injection of miR-34c lentivirus into mice caused repression of gastrocnemius muscle development. In summary, our data revealed that miR-34c can form a regulatory loop with *Notch1* to repress muscle development, and this result expands our understanding of muscle development mechanism.

## Introduction

Muscle satellite cells, also known as muscle stem cells, reside as quiescent cells beneath the basal lamina that surrounds muscle fibers, and function as myogenic precursors for muscle growth and repair^1, 2^. Quiescent muscle satellite cells are *PAX7* ^+^/*MYF5* ^−^, when exposed to stress or damage, they can respond and be activated^3, 4^. Activated muscle satellite cells are *PAX7* ^+^/*MYF5* ^+^, and after several rounds of replication, they will fuse to the nearby myofibers^5^. During these processes, the *Notch1* signaling pathway is known to play a critical role^6^.

Notch1 is a receptor that mediates intercellular signaling through a pathway conserved across the metazoan^7^. After two cleavages, the intracellular domain of Notch1 (N1ICD) is released and translocated into the nucleus, where it interacts with the CSL (CBF-1, Suppressor of hairless, Lag) family and forms part of a CSL-N1ICD complex on the DNA binding site (GTGGGAA) to regulate genomic DNA transcription^8, 9^. *Notch1* signaling is known to inhibit myogenic differentiation^10^ and has been proposed to promote the expansion of undifferentiated progenitors indirectly^11^. Rando *et al*. found that activation of *Notch1* signaling stimulates the proliferation of satellite cells and leads to the expansion of proliferating myoblasts. And, inhibition of *Notch1* signaling abolishes satellite cell activation and impairs muscle regeneration^11^. Also, recent studies found *Notch1* is active in quiescent muscle satellite cells, and *Notch1* signaling is critical for maintaining the quiescence of muscle satellite cells^12, 13^.

MicroRNA (miRNA) is a small non-coding RNA molecule (containing ~22 nucleotides) found in plants, animals, and some viruses^14^. MiRNA is encoded by nuclear DNA and usually transcribed by RNA polymerase II^15, 16^. As many as 40% of miRNA lie in the introns or even exons of other genes. Thus they usually are regulated together with their host genes. Other miRNAs are initially transcribed using their own promoter^16^. MiRNA exert their functions in RNA silencing by binding to complementary sequences within its target RNA^17, 18^. For example, in mice, miR-1 and miR-133 are clustered on the same chromosomal loci and transcribed together in a tissue-specific manner during development, but miR-133 enhances proliferation by repressing serum response factor, whereas miR-1 promotes myogenesis through repressing histone deacetylase 4^19^.

The miR-34 family members (miR-34a, miR-34b, and miR-34c) were discovered computationally^20^ and later verified by experiment^21, 22^. In human, three miR-34 precursors are produced from two transcriptional units, miR-34a precursor is transcribed from chromosome 1, and miR-34b and miR-34c precursors are co-transcribed from a region on chromosome 11^23^. But only miR-34a and miR-34c are found in pig^24, 25^. A previous study indicated miR-34a inhibits mouse smooth muscle cell proliferation by directly targeting *Notch1* ^26^. Another miR-34 family member miR-34c also has been shown to inhibit rat vascular smooth muscle cell proliferation^27^. But, the role of miR-34 plays in pig skeletal muscle development has not been reported.

Our laboratory has demonstrated that *Notch1* promotes PSCs proliferation^28^. We have also constructed an N1ICD overexpressing PSCs model for miRNA sequencing to examine satellite cell developmental mechanisms. From the RNA-seq data, we discovered that many miRNAs were differently expressed in the *N1ICD* overexpressing PSCs, including miR-34c^29^. However, there is no report regarding the role of miR-34c on PSCs development; therefore, the objective of this study was to define the role of miR-34c on PSCs development and ascertain whether there is a regulatory relationship between miR-34c and *N1ICD*.

In this study, we used miR-34c mimics and inhibitor to manipulate the miR-34c level in transfected PSCs and that elevated miR-34c not only inhibits PSCs proliferation but also promotes PSCs differentiation. Using dual-luciferase reporter assay and Chromatin immunoprecipitation (ChIP), we demonstrated there is a regulatory loop between *Notch1* and miR-34c. Finally, through miR-34c lentivirus injection into mice gastrocnemius muscle, we confirmed miR-34c represses mice muscle development. Our results not only established *Notch1* is the target gene of miR-34c but also discovered that *Notch1*, in turn, directly regulates miR-34c in PSCs. This reciprocal regulatory loop formed by miR-34c and *Notch1* that controls skeletal muscle development is novel, and this information expands our understanding of the mechanisms involved in muscle development.

## Results

### Overexpressing *N1ICD* decreases miR-34c expression *in vitro*

**Figure 1:**
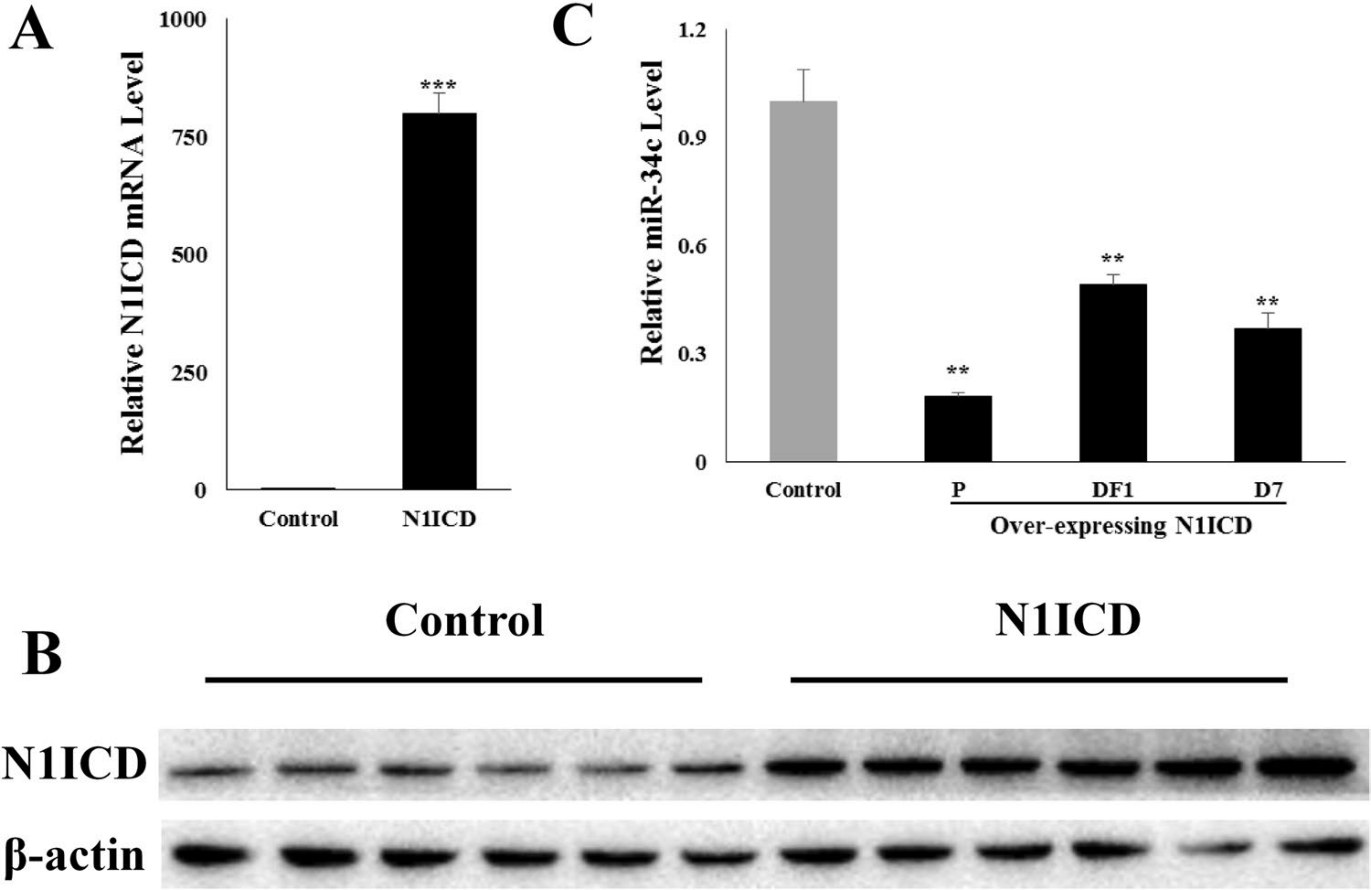
Overexpressing *N1ICD* decreases miR-34c expression during PSCs development. (**A**) PSCs treated with either pEGFP-*N1ICD* (*N1ICD*) or pEGFP-N1 (control). *N1ICD* mRNA level was increased in *N1ICD* overexpressing PSC on proliferation day 1 (P). (**B**) *N1ICD* protein level was increased in *N1ICD* overexpressing PSC on proliferation day 1. (**C**) qRT-PCR analysis of miR-34c on proliferation day 1 (P), differentiation day 1 (DF1) and differentiation day 7 (DF7), during the development of *N1IC*D-overexpressed PSCs. ^*^Indicates a difference between treatment and control. **p < 0.01; ***p < 0.001. The results are presented as Mean ± S.E.M. of three replicates for each group. The full-length blot images are presented in Supplementary Figure 1.

To explore the regulations of *Notch1* signaling in PSCs development, we constructed the constitutively activated *N1ICD* PSCs. Both the *N1ICD* mRNA and protein levels were increased (*p* < 0.001); Figs 1A and B) in the *N1ICD* overexpressed PSCs. The PSCs cell samples were collected on proliferation day 1 (P), differentiation day 1 (DF1) and differentiation day 7 (DF7). The expression of miR-34c was decreased in the *N1ICD* overexpressed cells (*p* < 0.01; Fig. 1C). These results demonstrate we have successfully overexpressed *N1ICD* in PSCs and N1ICD decreased miR-34c expression in all three periods of PSCs development.

### Overexpressing miR-34c inhibits PSCs proliferation *in vitro*

**Figure 2:**
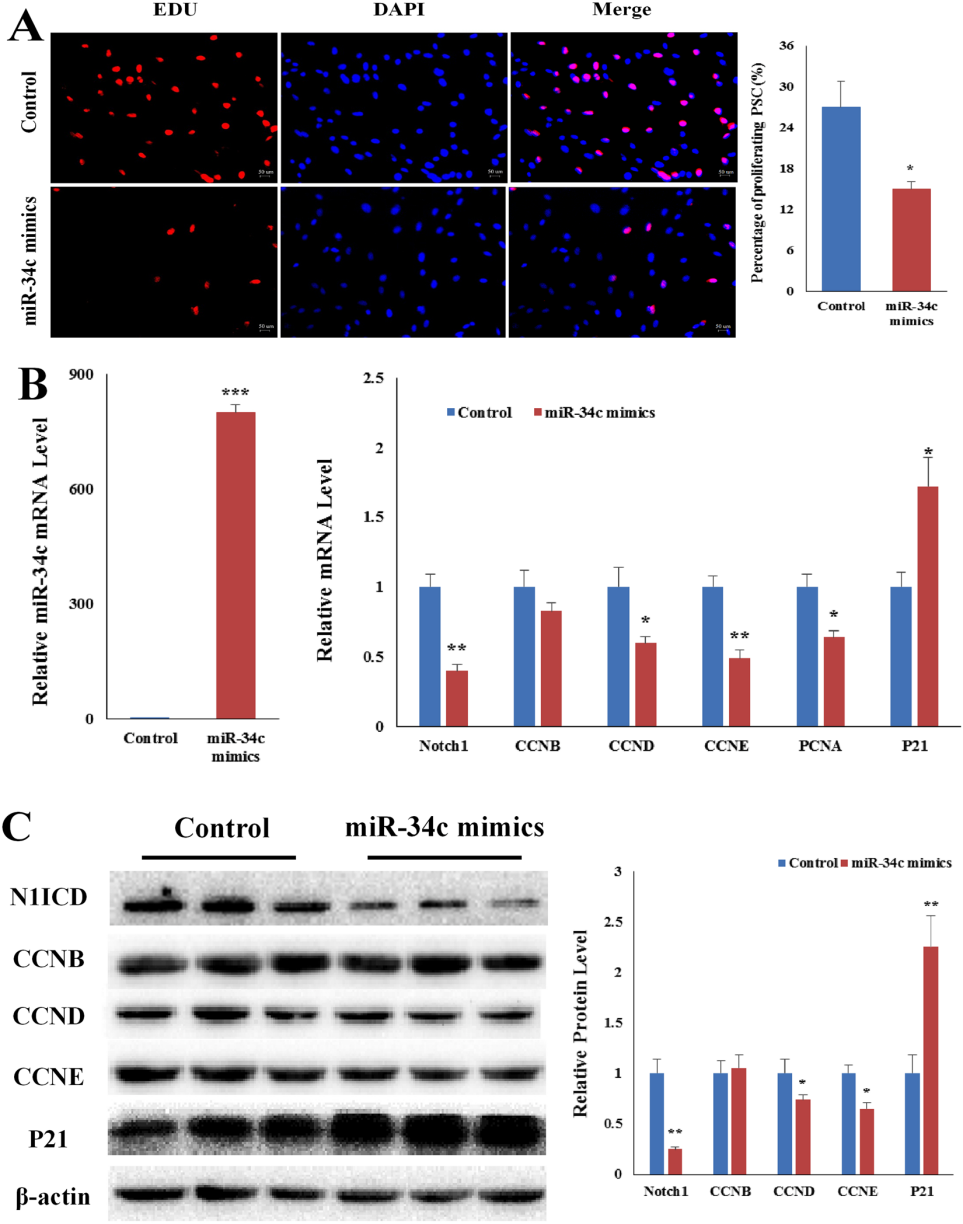
Overexpressing miR-34c mimics inhibits PSCs proliferation. (**A**) Overexpressing miR-34c in PSCs lowed PSCs proliferation. Representative images of the immunofluorescent staining for proliferating PSCs are shown. Proliferating PSCs were labeled with Edu fluorescent dye (red). (**B**) qRT-PCR confirmed miR-34c is negatively correlated with proliferation-related genes in PSCs transfected with miR-34c mimics. (**C**) Western blot result showed the protein level is corresponding to the qRT-PCR result. *p < 0.05; **p < 0.01. The results are presented as Mean ± S.E.M. of three replicates for each group. Magnification 200×. The scale bar on the photomicrographs represents 50 μm. The full-length blot images are presented in Supplementary Figure 2.

First, we investigated the role of miR-34 in PSCs proliferation. MiR-34c mimics was transfected into PSCs. Overexpressing miR-34c reduced the percentage of Edu positive cells (*p* < 0.05, Fig. 2A). qRT-PCR result showed miR-34c mimics increased (*p* < 0.001) cellular miR-34c level at 24 h after transfection (Fig. 2B). The miR-34c mimics decreased *Notch1, CCND, CCNE* and *PCNA* mRNA levels and increased *p21* mRNA level (*p* < 0.05, Fig. 2B). Western blot confirmed that miR-34c mimics reduced (*p* < 0.05) *Notch1*, *CCND* and *CCNE* protein level, and increased (*p* < 0.01) p21 protein level (Fig. 2C). However, miR-34c mimics had no effect on *CCNB* mRNA and protein levels (Fig. 2B and C).

**Figure 3:**
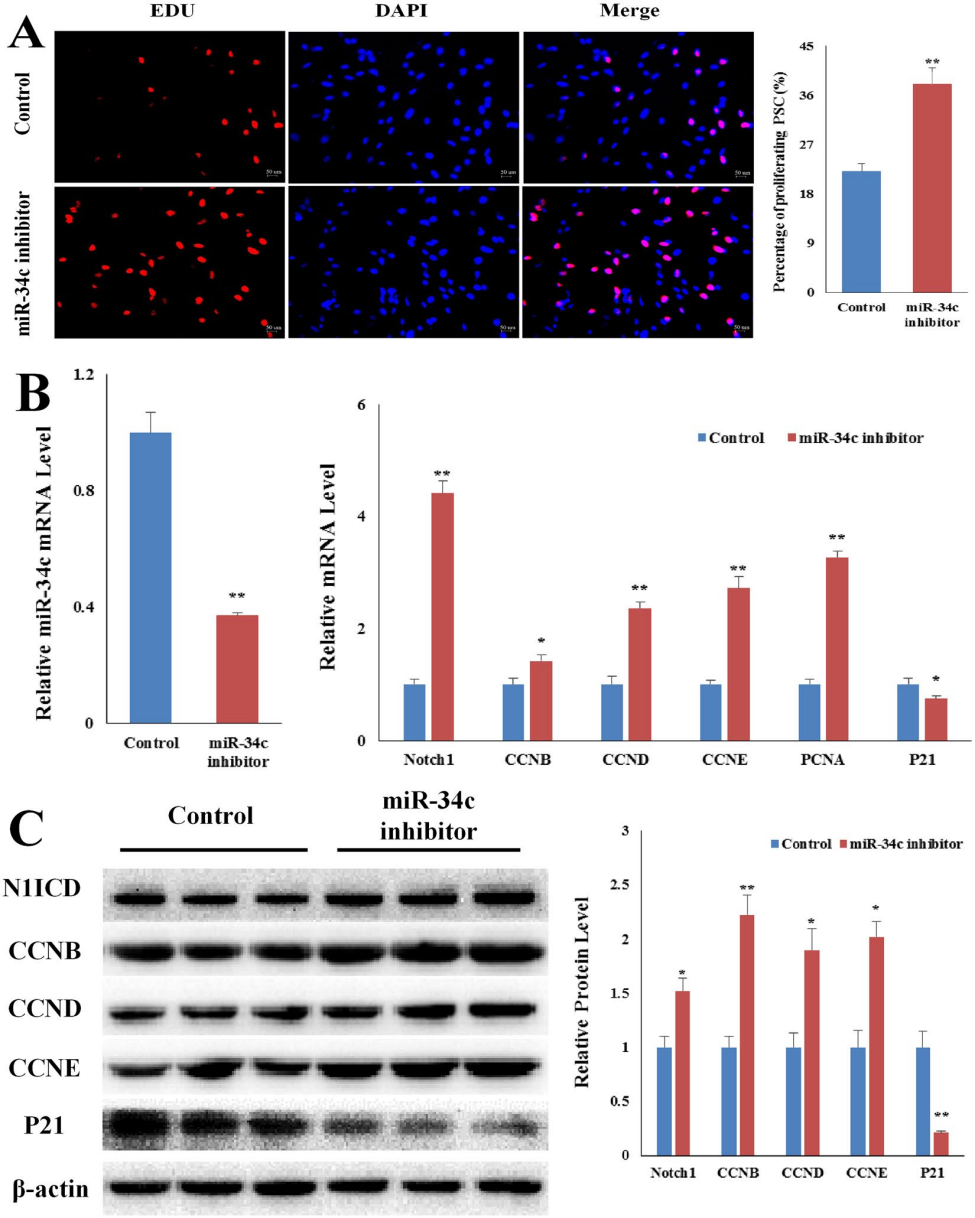
Overexpressing miR-34c inhibitor increase PSCs proliferation. (**A**) miR-34c inhibitor increased PSCs proliferation. Representative images of immunofluorescent staining of PSCs transfected with miR-34c inhibitor. (**B**) PSCs treated with either miR-34c inhibitor or Control confirmed miR-34c is negatively correlated with proliferation-related genes. (**C**) Western blot result showed protein level is corresponding to the qRT-PCR result. *p < 0.05; **p < 0.01. The results are presented as Mean ± S.E.M. of three replicates for each group. Magnification 200×. The scale bar on the photomicrographs represents 50 μm. The full-length blot images are presented in Supplementary Figure 2.

Next, we used miR-34c inhibitor to further ascertain the role of miR-34c on PSCs proliferation. MiR-34c inhibitor increased the percentage of Edu positive cells (*p* < 0.01, Fig. 3A). MiR-34c inhibitor reduced the endogenous miR-34c level (Fig. 3B). After transfected with miR-34c inhibitor, the mRNA level of *Notch1, CCNB, CCND* and *CCNE* and *PCNA* were increased and *p21* was decreased (*p* < 0.05, Fig. 3B). And the western blot result confirmed the qRT-PCR result, the protein level of *Notch1, CCNB, CCND* and *CCNE* were increased and *p21* was decreased (*p* < 0.05, Fig. 3C). Taken together, these results demonstrate miR-34c inhibits PSCs proliferation *in vitro*.

### Overexpressing miR-34c promotes PSCs differentiation

**Figure 4:**
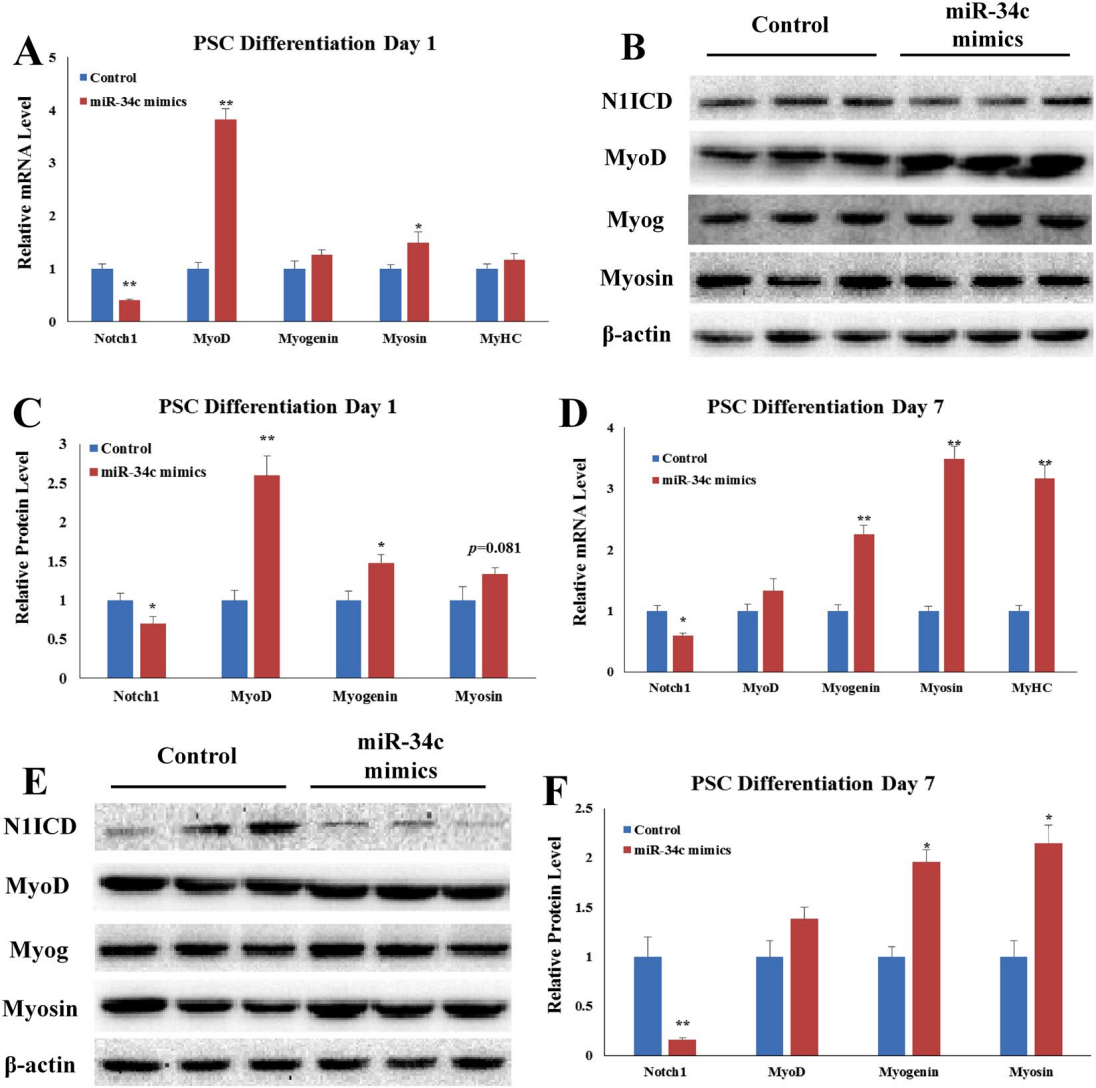
Overexpressing miR-34c mimics promotes PSCs differentiation. We measured the expression of *Notch1* and the myogenic marker genes after PSCs were transfected with miR-34c mimics. (**A**) qRT-PCR confirmed miR-34c increased mRNA level of myogenic marker genes on day 1 after differentiation induction. (**B**) We used western blot to measure protein level of *Notch1* and myogenic marker genes on day 1 after differentiation induction. (**C**) Western blot showed the protein level result is corresponding to the qRT-PCR result on day 1 after differentiation induction. (**D**) qRT-PCR confirmed miR-34c increased mRNA level of myogenic marker genes on day 7 after differentiation induction. (**E**) We used western blot to measure protein level of *Notch1* and myogenic marker genes on day 7 after differentiation induction. (**F**) Western blot showed the protein level result is corresponding to the qRT-PCR result on day 7 after differentiation induction. The results are presented as Mean ± S.E.M. of three replicates for each group. *p < 0.05; **p < 0.01. *Indicates a difference between Control and miR-34c mimics. The full-length blot images are presented in Supplementary Figure 3.

The medium was changed to differentiation medium for 1 and 7 days to study the function of miR-34c on PSCs differentiation. The expression of *Notch1*, *MyoD*, *myogenin*, *myosin*, and *MyHC* were measured. Overexpression of miR-34c reduced *Notch1* mRNA, and elevated *MyoD* mRNA and *myosin* mRNA on differentiation day 1 (*p* < 0.05, Fig. 4A). As for protein level of these genes, western blot result showed overexpression of miR-34c reduced *Notch1*, and elevated *MyoD* and *myogenin* on differentiation day 1 (*p* < 0.05, Fig. 4B). However, on differentiation day 7, overexpression of miR-34c showed no significant influence on *MyoD* mRNA and protein levels (Fig. 4C and D). The mRNA level of *myogenin*, *myosin*, and *MyHC* was elevated by the miR-34c overexpression on differentiation day 7 (*p* < 0.01, Fig. 4C), and protein level of *myogenin* and *myosin* follows the same pattern as the mRNA (*p* < 0.05, Fig. 4D).

**Figure 5:**
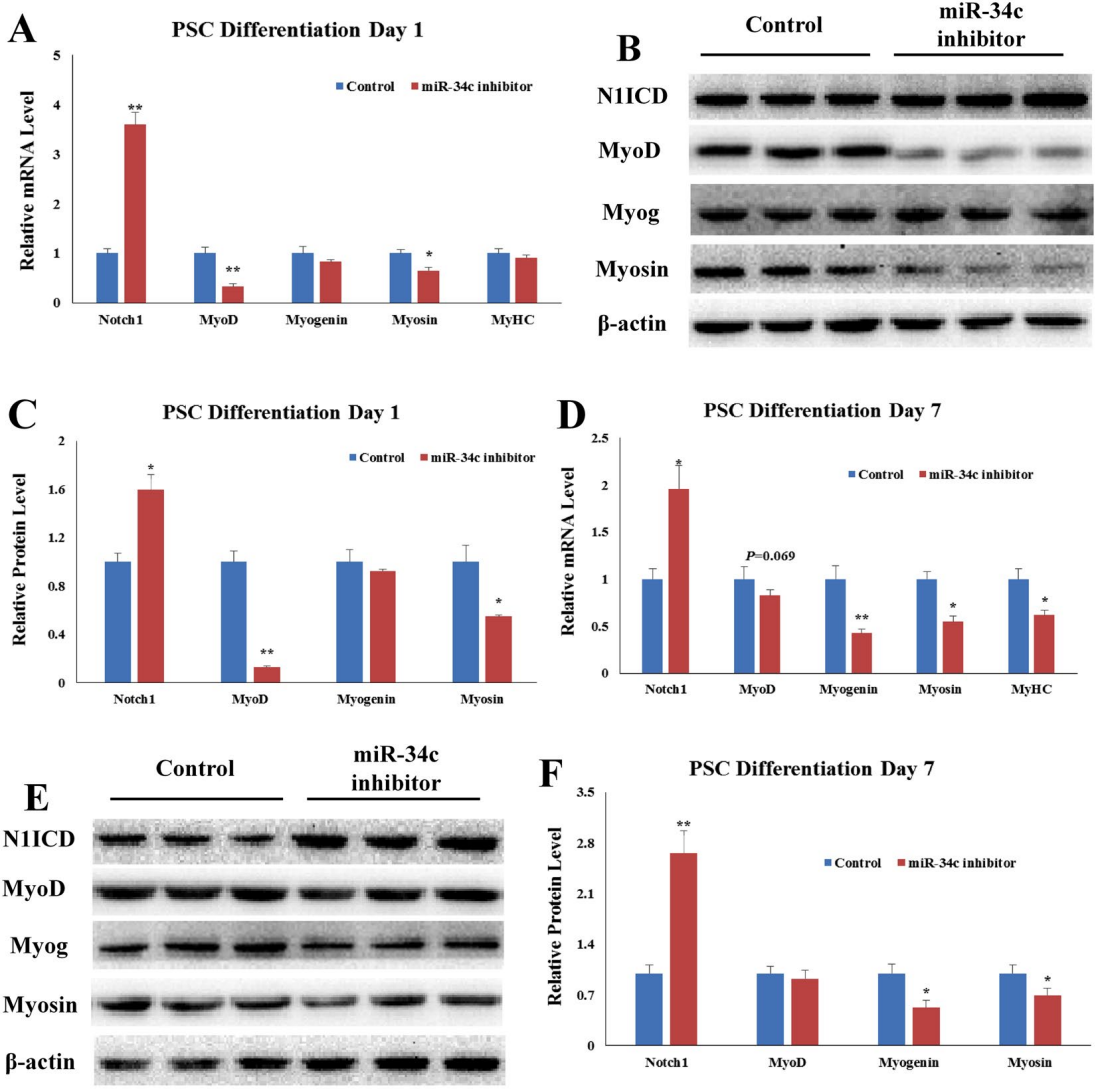
Overexpressing miR-34c inhibitor reduces PSCs differentiation. We measured the expression of *Notch1* and the myogenic marker genes after PSCs were transfected with miR-34c inhibitor. (**A**) qRT-PCR confirmed miR-34c inhibitor decreased mRNA level of myogenic marker genes on day 1 after differentiation induction. (**B**) We used western blot to measure protein level of *Notch1* and myogenic marker genes on day 1 after differentiation induction. (**C**) Western blot showed the protein level result is corresponding to the qRT-PCR result on day 1 after differentiation induction. (**D**) qRT-PCR confirmed miR-34c inhibitor decreased mRNA level of myogenic marker genes on day 7 after differentiation induction. (**E**) We used western blot to measure protein level of *Notch1* and myogenic marker genes on day 7 after differentiation induction. (**F**) Western blot showed the protein level result is corresponding to the qRT-PCR result on day 7 after differentiation induction. The results are presented as Mean ± S.E.M. of three replicates for each group. *p < 0.05; **p < 0.01. *Indicates a difference between Control and miR-34c mimics. MiR-34c mimics and Control are both synthetic oligonucleotide sequences; see Table S1 for details. The full-length blot images are presented in Supplementary Figure 3.

Next, miR-34c inhibitor or Control (miR-34c inhibitor and Control are both synthetic oligonucleotide sequences; see Table S1 for details) was transfected into PSCs, and the cells were induced to differentiation for 1 day and 7 days. PSCs transfected with miR-34c inhibitor increased the mRNA and protein levels of *N1ICD* on both differentiation day 1 and day 7 (Fig. 5). MiR-34c inhibitor decreased *MyoD* and *myosin* mRNA level on differentiation day 1 (*p* < 0.05, Fig. 5A). But miR-34c inhibitor had no effect (*p* > 0.05) on *myogenin* and *MyHC* mRNA level on differentiation day 1 (Fig. 5A). On differentiation day 1, western blot result shown miR-34c inhibitor decreased (*p* < 0.05) *MyoD* and *myosin* protein levels, but had no effect on myogenin (Fig. 5B). MiR-34c inhibitor decreased (*p* < 0.05) *myogenin*, *myosin* and *MyHC* genes mRNA level on differentiation day 7 (Fig. 5D). On differentiation day 7, western blot result shown miR-34c inhibitor decreased (*p* < 0.05) *myogenin* and *myosin* protein levels. But miR-34c inhibitor had no effect on *Myod* gene expression on differentiation day 7 (Fig. 5D and E). These results indicate elevated miR-34c promotes PSCs differentiation.

### Reciprocal regulation between *Notch1* and miR-34c

As overexpressing *N1ICD* decreases miR-34c expression during PSCs development (Fig. 1) and miR-34c mimics decreases *Notch1* gene expression in the proliferation period (Fig. 2), differentiation day 1and differentiation day 7 (Fig. 4), these results suggest a regulatory feedback may exist between *Notch1* and miR-34c.

**Figure 6:**
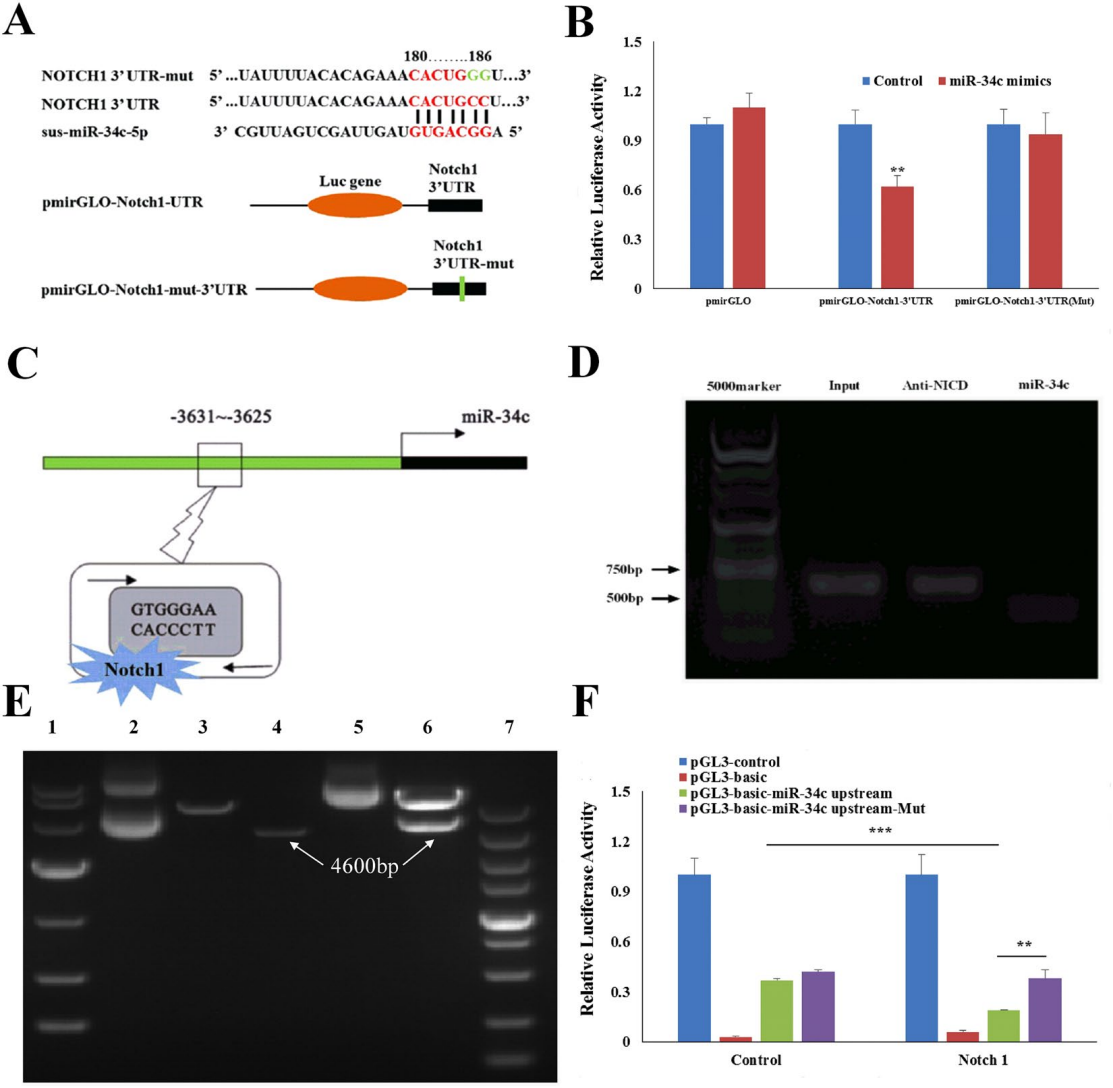
Negative feedback between miR-34c and *Notch1*. (**A**) Schematic representation of the sequence of *Notch1* 3′UTR, *Notch1* 3′UTR-mut, miR-34c, and luciferase reporter constructs. (**B**) Luciferase reporters shown in (**A**) were transfected into HEK-293T cells with either miR-34c mimics or Control respectively. Luciferase activity was determined 24 h after transfection. (**C**) Schematic representation of Notch1 binds at the upstream of miR-34c in the genome. (**D**) PSCs transfected with pEGFP-*N1ICD* and were cultured in growth medium for 24 h. These PSCs were immune precipitated with the Notch1 antibody. The precipitated DNA was used to amplify the fragments of -3631 upstream of miR-34c gene by primers (arrows). (**E**) Cloned miR-34 upstream of its genomic site (about 4600 bp, the fourth lane), and constructed pGL3-basic-miR-34 upstream recombinant vector (the fifth and sixth lanes). Lanes 1–7 represent DL 10000, pGL3-basic, double digested pGL3-basic, double digested miR-34 upstream of its genomic site, pGL3-basic-miR-34 upstream recombinant vector, double digested pGL3-basic-miR-34 upstream recombinant vector, DL5000, respectively. (**F**) N1ICD decreased the pGL3-basic-miR-34 upstream recombinant vector relative luciferase activity. N1ICD with pGL3-basic-miR-34 upstream recombinant vector relative or pGL3-basic-miR-34 upstream (mut) recombinant vector were transfected into HEK-293T cells respectively. Luciferase activity was determined 24 h after transfection. *p < 0.05; **p < 0.01; ***p < 0.001; Student’s *t-test*. The results are presented as Mean ± S.E.M. of three replicates for each group.

Since *N1ICD* expression was decreased after PSCs transfected with miR-34c mimics (Figs 2 C and 4B), *Notch1* may be a potential target gene of miR-34c. A dual-luciferase reporter assay was used to identify the miR-34c binding sites on *Notch1*-3′UTR (Fig. 6A). We found relative luciferase activity was decreased (*p* < 0.01; Fig. 6B) when HEK-293T cells were co-transfected with miR-34c mimics and pmirGLO-*Notch1*-3′UTR. However, mutated pmirGLO-*Notch1*-3′UTR did not change relative luciferase activity (Fig. 6B). These results suggest that *Notch1* is the direct target gene of miR-34c.

As the miR-34c level is reduced when *N1ICD* was overexpressed during PSCs development (Fig. 1A) and a CSL-N1ICD complex binding site (GTGGGAA) exists at upstream of the miR-34c genomic site (Fig. 6C), we measured the DNA fragment binding with CSL-N1ICD complex by ChIP. A fragment about 400 bp long located at -3631 upstream of miR-34c was amplified (Fig. 6D). The CSL-N1ICD complex binds directly to the -3631~-3625 sites of miR-34c. This result indicates CSL-N1ICD complex may regulate miR-34c transcription. So we constructed the pGL3-basic-miR-34 upstream recombinant vector (pGL3-basic-miR-34 upstream). As shown in Fig. 6E the miR-34 upstream of its genomic site (about 4600 bp) is inserted into the pGL3-basic vector. Through the dual-luciferase reporter assay, we found N1ICD decreased the pGL3-basic-miR-34 upstream recombinant vector relative luciferase activity, but this inhibition was abolished by the mutated CSL-N1ICD complex binding site (GTGGGAA) (Fig. 6F). These findings establish there exists a regulatory loop between *Notch1* and miR-34c.

### MiR-34c represses muscle development *in vivo*

**Figure 7:**
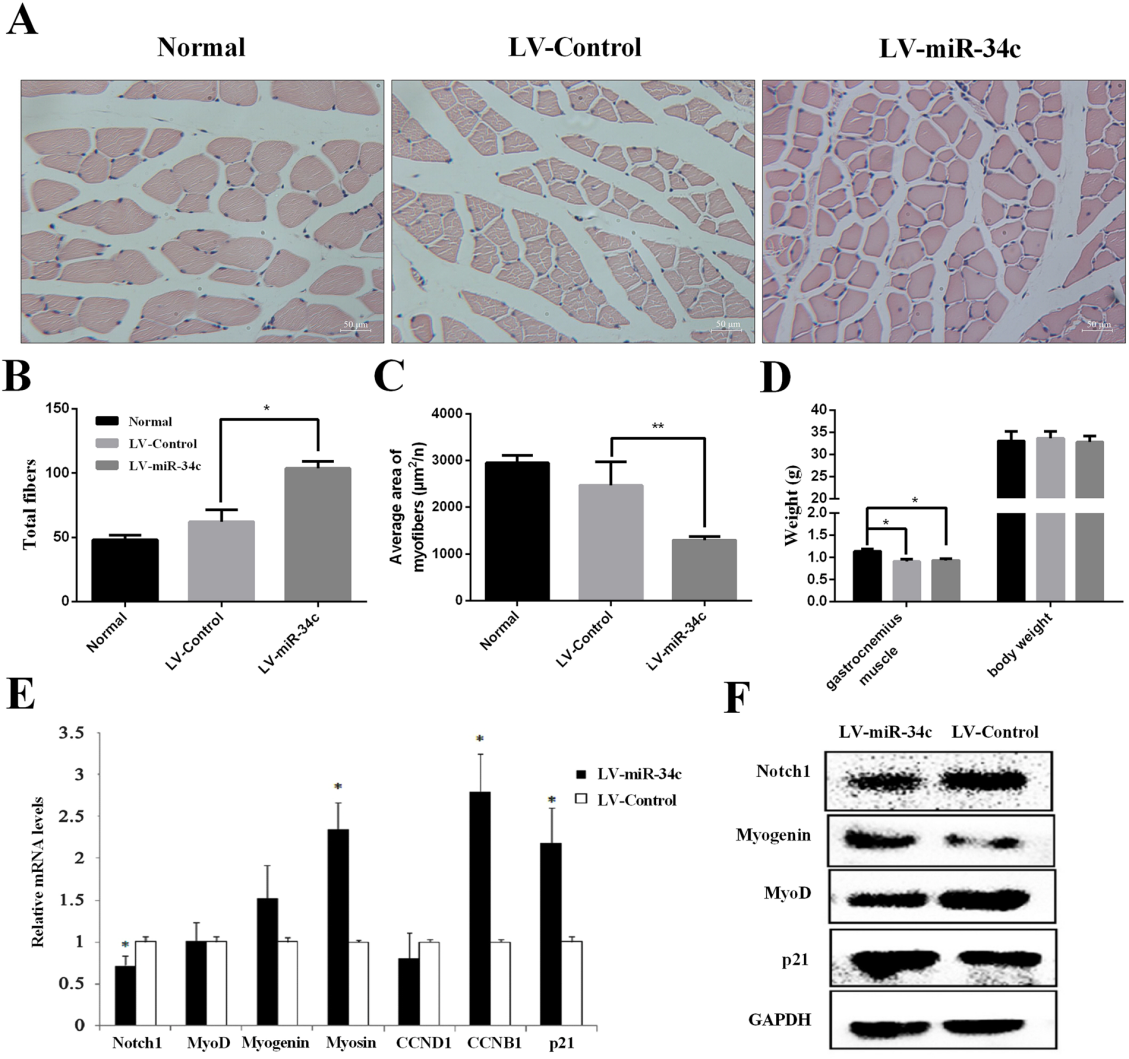
MiR-34c injection represses muscle development. (**A**) H&E stained cross-section of the gastrocnemius muscle (GM) from Normal (physiological saline), LV-Control and LV-miR-34c. MiR-34c or microRNA-control was delivered by a lentiviral vector (LV). (**B**). Total fibers in one field were increased after injection with LV-miR-34c. (**C**) The average area of muscle fiber cross-sections was decreased after injection with LV-miR-34c. (**D**) Body weight and gastrocnemius muscle weight seven days after LV-Control or LV-miR-34c injection, respectively. (**E**) qRT-PCR analysis of *Notch1*, myogenic markers, cell cycle proteins, and p21 in the gastrocnemius muscle after LV-Control or LV-miR-34c injection for 7 days. (**F**) Western blots of proteins isolated from gastrocnemius muscles, which were injected either LV-Control or LV-miR-34c after day 7, respectively. The results are presented as Mean ± S.E.M. of three replicates for each group. *p < 0.05, **p < 0.01; *Indicates differences between LV-Control and LV-miR-34c in mice gastrocnemius muscle except the Fig. 6D. In Fig. 6D *Indicates differences between Normal and LV-Control or Normal and LV-miR-34c in mice gastrocnemius muscle. Magnification 200×. The scale bar on the photomicrographs represents 50 μm.

To evaluate the function of miR-34c *in vivo*, we injected lentivirus expressing miR-34c mimics or Control into mice gastrocnemius muscle. Both miR-34c mimics and Control are synthetic oligonucleotide sequences delivered by the lentiviral vector. Body weight and characteristics of gastrocnemius muscle were recorded, and total RNA and protein were extracted a week later. Total myofibers in a single field were increased (*p* < 0.01, Fig. 7A,B) in the LV-miR-34c injected group compared with the Normal group and LV-Control (*p* < 0.05, Fig. 7A,B). Consistent with this result is the average area of myofibers decreased (*p* < 0.01, Fig. 7C), which means miR-34c repressed muscle development *in vivo*. Body weights were no difference among all groups, but the weights of gastrocnemius muscle were decreased in the LV-miR-34c treatment group and Normal groups (*p* < 0.05, Fig. 7D). After injecting LV-miR-34c, *MyoD* protein level was decreased (Fig. 7F) and *myogenin* protein level was increased (Fig. 7F), but their mRNA levels were not different (Fig. 7E). LV-miR-34c injection also increased *myosin* mRNA level (*p* < 0.05, Fig. 7E). *CCND1* mRNA level was decreased after LV-miR-34c injection, but mRNA levels of *CCNB1* and *p21* were increased (*p* < 0.05, Fig. 7E). Meanwhile, p21 protein level was also increased (Fig. 7F). *Notch1* mRNA level was decreased in the LV-miR-34c injection group (*p* < 0.05, Fig. 7F). *Notch1* protein level was also decreased (Fig. 7F). These results demonstrate that injecting LV-miR-34c miR-34c represses muscle development *in vivo*.

## Discussion

Skeletal muscle is the major component of lean body mass in livestock. Postnatal muscle growth mainly depends on the hypertrophy of existing muscle fibers, and muscle satellite cells have been shown to be responsible for this process^30^. The development of muscle satellite cells is affected by many factors. The microenvironment or niche where muscle satellite cells locate is relative stable. The niche contains extracellular matrix proteins, vasculature system, muscle residence cells and muscle fibers^31^. However, the homeostasis of the niche is broken under stress or injury. Heat stress is well established to affect porcine growth rate negatively. Heat stress produces cellular stress and changes the rates of PSCs proliferation, protein turnover and abundance of heat shock proteins (Hsp) mRNA and protein in PSCs cultures^32^. Furthermore, the activity of PSCs changes with age. Mesires *et al*.^33^ isolated PSCs from the hind limb muscles of pigs at 1, 7, 14, and 21 weeks of age. The number of PSCs in these muscles decreased from 30% at one week to 14% at seven weeks of age and remained at a constant level after week 14. That may reflect a decrease in the number of differentiating PSCs or accelerated fusion into myofibers^33^.

*Notch* signaling has been shown to stimulate the proliferation and inhibit the differentiation of muscle satellite cells^34^. Wen *et al*.^6^ used mice skeletal muscle satellite cells as a model and constructed a myogenic cell lineage in which *N1ICD* was constitutively activated. The CSL-N1ICD complex binds to the DNA binding site (GTGGGAA) upstream of PAX7 and promotes the “self-renewal” of muscle satellite cells^6^. Consequently, we would like to know whether CSL-N1ICD complex binds to the binding site upstream of any miRNAs to affect its transcription. The miRNAs are critical regulators of many biological processes by modulating expression of genes at the post-transcriptional level. Proliferation and differentiation are mutually exclusive during myogenesis, and miRNAs are critically involved in balancing these two processes^35^. Many miRNAs, such as miR-1, miR-133, miR-29, miR-214, miR-206, miR-486, miR-208b, and miR-499 were involved in the regulation of skeletal myogenesis by binding to its target genes^36, 37^. To establish *N1ICD* regulates miR-34c expression, *N1ICD* was transfected into PSCs in primary culture. *N1ICD* was constitutively activated in these transfected PSCs. These PSCs were used to conduct our *in vitro* studies. Using ChIP assay, we showed that CSL-N1ICD complex binds directly to the −3631~−3625 sites of miR-34c. We confirmed the N1ICD reduces the transcriptional activity of the miR-34c promoter through dual-luciferase reporter assay. We have also established *Notch1* is a direct target gene of miR-34c using a dual-luciferase reporter assay. These results demonstrate there exists a regulatory loop between *Notch1* and miR-34c in PSCs development.

Postnatal muscle development involves muscle satellite cells activation, re-entering the cell cycle for replication, and subsequently differentiation into myoblasts^38, 39^. MiR-34c has been shown to be a new modulator of VSMC proliferation through targeting *SCF* ^27^. But, smooth and skeletal muscle tissues are composed of distinct cell types^40^. These two muscle cell types are also believed to have distinct embryonic origins. Smooth and skeletal muscle cells express distinct isoforms of the structural genes used for contraction^41^. To our knowledge, there is no report about the miR-34c function on skeletal muscle development. Our study ascertains that miR-34c inhibits PSCs proliferation by inhibiting *Notch1* expression. PSCs proliferation is dependent on an active cell cycle. In the cell cycle, CCND is the first cyclin produced; thus, we measured *CCND* expression as the PSCs proliferation marker. CCND binds to existing CDK4, forming the active CCND-CDK4 complex. Then, CCND-CDK4, in turn, phosphorylates the retinoblastoma susceptibility protein (Rb). Rb dissociates from the E2F/DP1/Rb complex, activating E2F. E2F promotes various genes such as *CCNE* and *DNA polymerase* transcription. Therefore, we also measured *CCNE* expression as the PSCs proliferation marker. CCNE binds to CDK2, forming the CCNE-CDK2 complex, which pushes the cell from G_1_ to S phase. CCNB-CDK1 complex activation causes breakdown and prophase initiation, subsequently, CCNB-CDK1 deactivation causes the cell to exit mitosis^42–44^. The *p21* protein is a cell cycle regulator, and it binds to cyclin-CDKs complexes and inhibits their activities^45, 46^. Thus, *CCNB* and *P21* were also used as the PSCs proliferation markers in our study. In our study, we used Edu assay to confirm miR-34c inhibits PSCs proliferation, and our qRT-PCR and western blot results show miR-34c is positively correlated with *p21*, and negatively correlated with *CCNB*, *CCND*, and *CCNE*.

For PSCs differentiation, *MyoD* is an early myogenic marker^47^, and *myogenin* and *MyHC* are terminal differentiation markers^48, 49^. Thus, in our study, *MyoD* and *myogenin*/*MyHC* showed different expression patterns on differentiation day 1 and day 7.

The *in vitro* study is commonly used as an experimental model for the animals. However, results obtained using *in vitro* studies need to be verified by the *in vivo* studies to confirm the validity of the *in vitro* data. Thus, to ascertain the miR-34c function observed in our cell culture studies, we conducted the *in vivo* study in mice. We injected miR-34c into the gastrocnemius muscle of the mice to establish the changes in muscle growth and gene expressions. Since the mice used in this study were 4-week-old, they were in rapid growing stage. Thus the presence of virus affected muscle development, which explains the gastrocnemius muscle weight of the injected virus groups (LV-Control and LV-miR-34c) was lower than that of the Normal group. After birth, the skeletal muscle myofiber number remains constant, and muscle growth depends on the satellite cell proliferation and fusion to the existing myofibers^50–52^. But miR-34c inhibited the skeletal muscle satellite cell proliferation by targeting *Notch1*, and miR-34c reduced the number of satellite cells to be fused to existing myofibers, resulting in smaller muscle fiber diameter after miR-34c injection.

This study would be better to have the miR-34c inhibitor group in the *in vivo* study. However, after considerable deliberations, we decided to forgo the miR-34c inhibitor group for the following reason: the mice used in this study are 4-week-old. Mice at this age most likely are in the rapid muscle-growing stage. Therefore, injection of LV-miR-34c inhibitor may not obtain additional muscle growth; thus, the value of using more mice may be questionable following the principle of a minimal number of animals to be used in any experiments. Also, the experimental cost was considerably lower with fewer animals used. Since our *in vitro* study has shown that overexpressing miR-34 inhibits muscle development, we believe the miR-34c overexpression experiment alone would be sufficient to demonstrate the role of miR-34c *in vivo*. Furthermore, the objective of this particular experiment was to ascertain whether the miR-34c inhibits muscle development *in vivo*. The postnatal muscle development involves muscle satellite cells activation, re-entering the cell cycle for replication, and subsequent differentiation into myoblasts. In this study, the gastrocnemius muscles were dissected one week after injection; the activated PSCs would have already differentiated into myoblasts. Thus, muscle fiber diameter is the most intuitive phenotypic data for muscle development. Therefore, we did not conduct additional immunostaining of any myogenic markers in the mice gastrocnemius muscle.

In conclusion, our research demonstrates that miR-34c inhibits PSCs proliferation but promotes PSCs differentiation *in vitro*, and miR-34c represses pig muscle development *in vivo*. These results expand our understanding of muscle development mechanism in which miRNAs and genes participate in a regulatory loop that controls skeletal muscle development. Furthermore, this finding has implications for both human health and animal breeding.

## Materials

All animals used in this study were with the approval of the College of Animal Science, South China Agricultural University. All experiments were conducted following ‘the instructive notions with respect to caring for laboratory animals’ issued by the Ministry of Science and Technology of the People’s Republic of China.

### Isolation and culture of primary porcine satellite cells (PSCs)

Three one-day old male Landrace piglets (approximately 1.5 kg) were anesthetized using sodium pentobarbital, and muscles from legs were used for PSCs isolation. The PSCs isolation method used has been described previously^28, 53^. When the PSCs reached approximately 80–90% confluent, cells were allowed to differentiate in DMEM/F12 with 2% horse serum, 100 U/mL penicillin, and 100 μg/ml streptomycin (GIBCO BRL) for 1 day or 7 days. After differentiation, the cells were washed twice with cold phosphate-buffered saline (PBS) before protein extraction or total RNA extraction.

### PSCs transfection

PSCs were seeded in 6-well plates (2 × 10^5^ cells per well). PSCs transfected with pCDNA3.1-*N1ICD*, miR-34c inhibitor, miR-34c mimics or Control by Lipofectamine 2000 (Invitrogen, Carlsbad, CA, USA), according to the manufacturer’s instructions. After 6 hours, the medium was replaced with new growth medium and cells were maintained in growth medium for an additional 12 h before myogenic differentiation induction. MiR-34c mimics and inhibitor were (see Table S1) purchased from GENEWIZ (Suzhou, China).

### RNA extraction and PCR analysis

Total RNAs were extracted from muscles or porcine satellite cells using TRIzol reagent (Invitrogen, Carlsbad, CA, USA) according to the manufacturer’s instructions. After digesting with DNase I (Takara Bio Inc., Japan), total RNAs (0.5 μg) were reverse transcribed to cDNA using PrimeScript^TM^ RT Master Mix (TaKaRa, Otsu, Shiga, Japan) according to the manufacturer’s instructions. SYBR Green Real-time PCR Master Mix reagents (Toyobo Co., Ltd., Osaka, Japan) were used for real-time quantitative polymerase chain reaction (PCR), and PCR reactions carried out on a CFX96™ Optical Reaction Module (BIO-RAD, USA). The relative expression of mRNAs and microRNA were normalized with *GAPDH* or *U6* levels using the ΔΔ^*Ct*^ method^54^. ^ΔΔ^Ct is defined as the ratio of the relative mRNA level of the target gene between the experimental group and the control group. Primers were designed using Primer Premier 5 according to the pig genes sequence obtained from NCBI. Primers used for PCR are shown in Table S1.

### Edu labeling

PSCs transfected with miR-34c mimics, miR-34c inhibitor or Control and maintained in growth medium. Twenty-four hours later, these PSCs were used for Edu labeling by Cell-Light™ Edu Apollo®488 *In Vitro* Imaging Kit (RiboBio, Guangzhou, China) according to the manufacturer’s instructions. The Edu labeled PSCs were washed with PBS, then observed and recorded using a Nikon TE2000-U inverted microscope (Nikon Instruments, Tokyo, Japan). The Edu positive PSCs were counted using Image Pro Plus (Media Cybernetics, Inc., Silver Spring, MD, USA).

### Western blot analysis

PSCs and mice gastrocnemius muscle were lysed in RIPA buffer containing 1 μM PMSF. About 20 μg protein lysates were separated with SDS-PAGE and then electroblotted onto polyvinylidene fluoride membranes (BIO-RAD, USA). The membranes were blocked at room temperature for 2 hours and incubated with different diluted antibodies at 4 °C overnight. Finally, the polyvinylidene fluoride membranes were incubated with horseradish peroxidase-conjugated secondary antibodies at room temperature for 40 minutes. The antibodies used in this study are listed in Table S2.

### Luciferase Reporter Assay

*Notch1* 3′ UTR sequence was amplified and inserted into pmirGLO Vector (Ambion, Carlsbad, CA, USA). For the luciferase reporter assay, HEK 293 T cells were co-transfected with pmirGLO-*Notch1*-3′UTR plus either miR-34c mimics or Control for 48 hours. Both pmirGLO and pmirGLO-*Notch1*-3′UTR-mut were used as the controls for pmirGLO-*Notch1*-3′UTR. The activities of firefly and Renilla luciferases were determined using the Dual-Luciferase Reporter Assay System (Promega, Madison, WI, USA), and firefly luciferase activity was normalized to that of Renilla luciferase.

A ~4600 bp sequence upstream of miR-34c in genomic DNA was amplified and inserted into pGL3-basic Vector (Ambion, Carlsbad, CA, USA). For the luciferase reporter assay, HEK 293 T cells were co-transfected with pGL3-basic-miR-34c upstream, pRL-TK plus either pCDNA3.1-*N1ICD* or pCDNA3.1. Either pGL3-basic-miR-34c upstream-mut or pGL3-control was used as a control for pGL3-basic-miR-34c upstream. The activities of firefly and Renilla luciferases were determined using the Dual-Luciferase Reporter Assay System (Promega, Madison, WI, USA), and firefly luciferase activity was normalized to that of Renilla luciferase.

### ChIP assay

The ChIP-IT^®^ Express Magnetic Chromatin Immunoprecipitation Kit & Sonication Shearing Kit (Catalog Nos 53008) was purchased from Active Motif (Carlsbad, CA). For ChIP assay, the DNA was immunoprecipitated with the anti-N1ICD antibody (1:1000 dilution, Cell Signaling Technology, Danvers, MA, United States), and ChIP analysis was performed according to the manufacturer’s protocol. Primers used for ChIP assay are shown in Table S1. DNA samples before immunoprecipitation were used as a template for input control.

### Tissue sampling and H&E

Mice were purchased from Guangdong Medical Lab Animal Center, and lentivirus containing miR-34c mimics or Control were purchased from Shanghai JiKai Gene Chemical Technology Co., LTD. Nine mice were divided into three groups, and each group contained three mice. Mice were injected with physiological saline, LV-Control or LV-miR-34c. Gastrocnemius muscles were dissected from each mouse one week after injection for extracting total RNA and proteins. Mice gastrocnemius muscles were stained with Hematoxylin and Eosin (H&E), and the cross section area of individual myofibers was photographed using Nikon TE2000-U inverted microscope (Nikon Corporation, Tokyo, Japan) and measured using Image-Pro Plus (IPP) 6.0 software (Media Cybernetics, Inc., Silver Spring, MD, USA).

### Statistical analysis

All data are expressed as the mean ± standard error of the mean (S.E.M.). For all the experimental data in this study, including miR-34c inhibiting PSCs proliferation, miR-34c promoting PSCs differentiation and miR-34c repressing mice muscle development, we are comparing only the control group to the treatment group; thus we used the student’s t-test for the statistical analyses (SPSS 18.0, Chicago, IL, USA).

### Data availability statement

All data generated or analyzed during this study are included in this published article (and its Supplementary Information files).

## Electronic supplementary material

Supplementary Information
