# Supplementary material for: MiR-34c represses muscle development by forming a regulatory loop with Notch1
Source: Sci Rep. 2017 Aug 24;7:9346. doi: 10.1038/s41598-017-09688-y (PMC5571228; doi:10.1038/s41598-017-09688-y)
Supplement: Supplementary file 1 — Supplementary Information [file 41598_2017_9688_MOESM1_ESM.pdf]

MiR-34c represses muscle development by forming a regulatory loop with *Notch1*

Lianjie Hou[#1], Jian Xu[#1], Huaqin Li[1], Jinxin Ou[1], Yiren Jiao[1], Chingyuan Hu[2], Chong Wang[*1]

Supplementary Information

Supplementary figures and legends

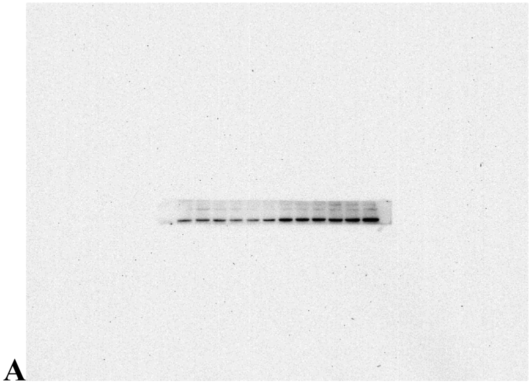
A

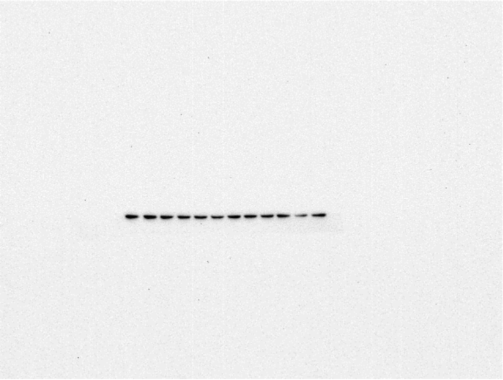
B

Supplementary Figure 1. Full length blots used for Figure 1B. A: N1ICD; B: β-actin. In all figures lanes 1-6, and 7-12 represent PSCs transfected with pCDNA3.1-N1ICD and pCDNA3.1, respectively.

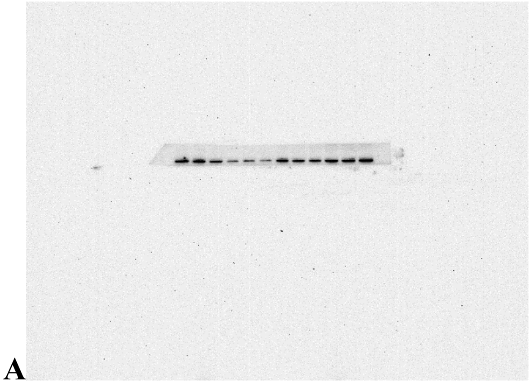
A

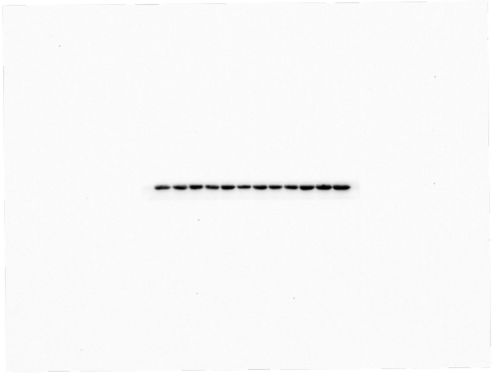
B

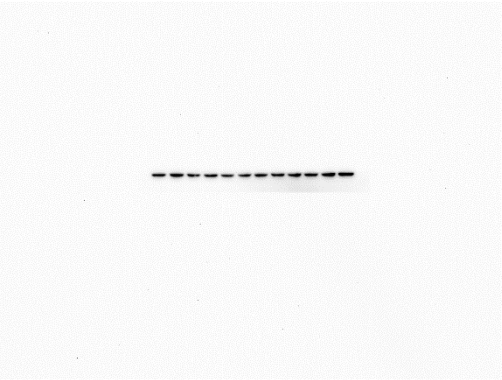
C

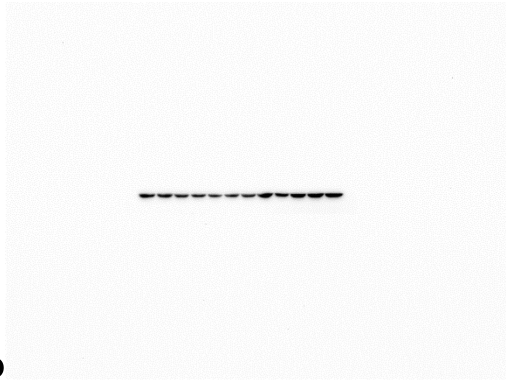
D

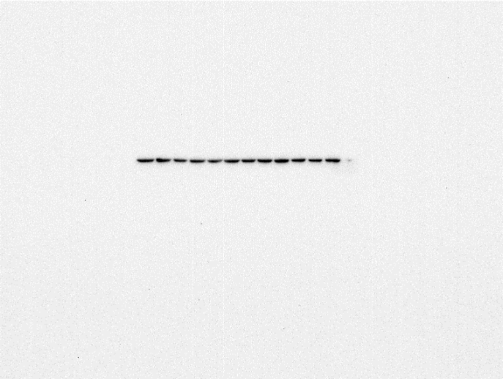

E

Supplementary Figure 2. Full length blots used for Figure 2C and Figure 3C. A: N1ICD; B: CCNB; C: CCND; D: CCNE; E: p21; D: β-actin. In all figures lanes 1-3, 4-6, 7-9, and 10-12 represent PSCs transfected with miR-34c mimics control, miR-34c mimics, miR-34c inhibitor control and miR-34c inhibitor, respectively.

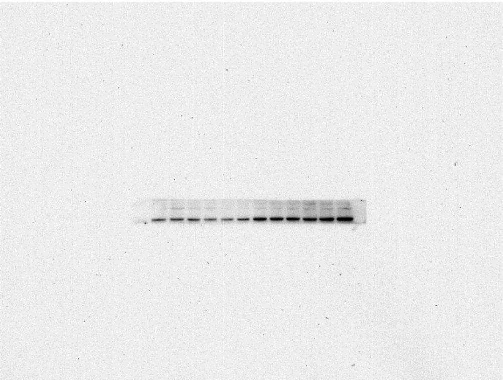

A

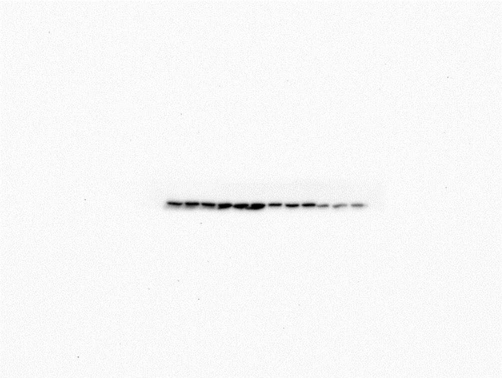

B

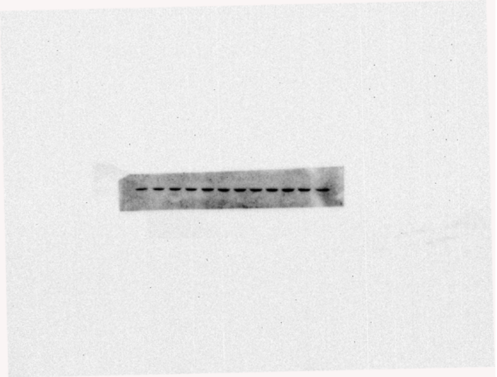

C

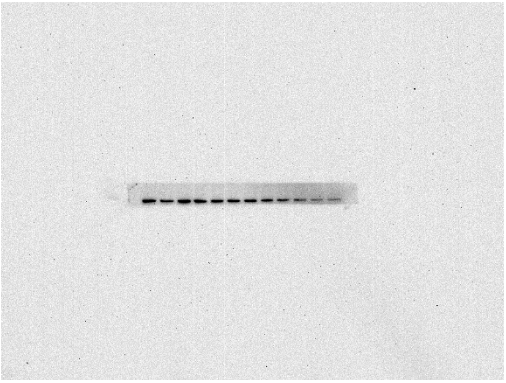

D

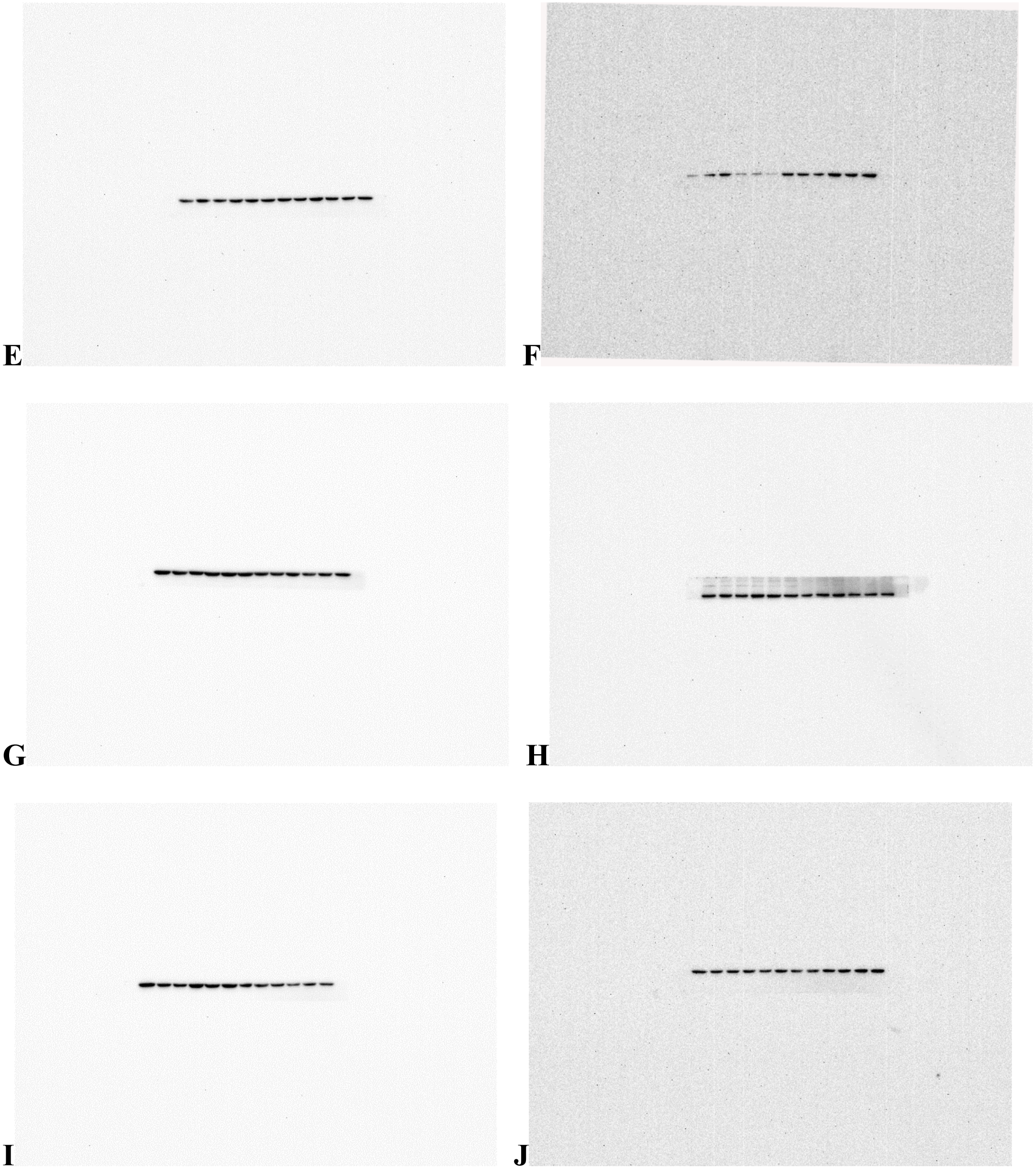

Supplementary Figure 3. Full length blots used for Figure 4B, Figure 4E, Figure 5B and Figure 5E. A: N1ICD on differentiation day 1; B: CCNB on differentiation day 1; C: CCND on differentiation day 1; D: CCNE on differentiation day 1; E: β-actin on differentiation day 1; F: N1ICD on differentiation day 7; G: CCNB on differentiation day 7; H: CCND on differentiation day 7; I: CCNE on differentiation day 7; J: β-actin on differentiation day 7. In all figures lanes 1-3, 4-6, 7-9, and 10-12 represent PSCs transfected with miR-34c mimics control, miR-34c mimics, miR-34c inhibitor control and miR-34c inhibitor, respectively.

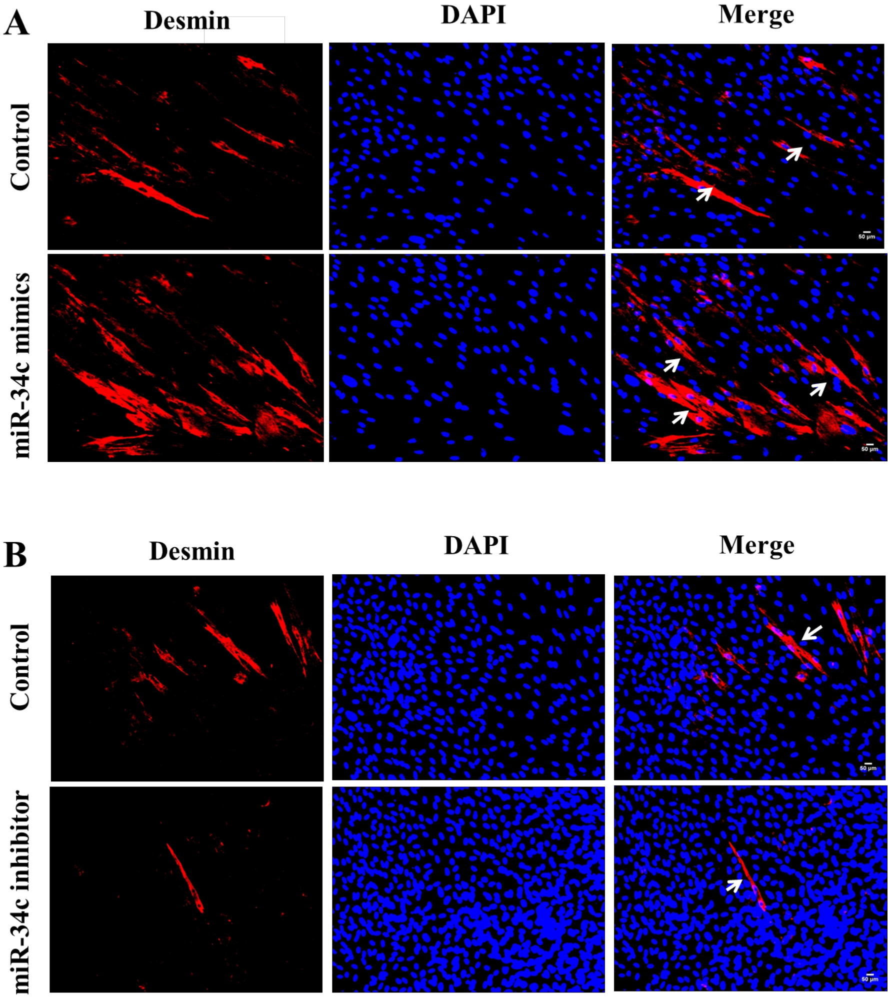

Supplementary Figure 4. MiR-34c promotes PSCs to form myotubes. MiR-34c mimics increased the percentage of desmin positive PSCs and miR-34c inhibitor decreased the percentage of desmin positive PSCs. Representative images of the immunofluorescent staining for differentiated PSCs are shown. Desmin: red, a molecular marker of myogenesis; DAPI: blue, cell nuclei. N = 3 for each treatment. Magnification 200×.

Supplementary tables

Table S1. Primers Used for PCR Analyses.

Gene name	Forward primer sequence (5'-3')	Reversed primer sequence (5'-3')
N1ICD	GTCTACGCTAATCTCA	GTCTGCGGCTCCTCCTTCA
Notch1	GGTCACCGCCAAAGTCCG	CAGCCCTCTGTAGTGCTTCATT
CCNB	GGATCACCAGGAACACG	TTCCAGAGGTTTCGGTAG
CCND	TGAGGAGCAGAAGTGCGAAGA	GGCAGTCAAGGGAATGGT
CCNE	TGAGGAGCAGAAGTGCGAAGA	GGAGGGTGGGTTGGAAAT
PCNA	CGGATGGCTGTCATTGGG	CACTGGAAGGCGAAGGTTTT
P21	TCTGCCAAGAGGGCTAAGTT	CTCAGGCGTTCCAAGACATT
MyoD	GAGAACAATCAGGGAGAAAG	TTGGTGGGTATGTCATCAGC
Myogenin	TAAGCACCCAGCAACGACATA	GACAGAATCCGAATCACTCC
Myosin	CATCTTCTCATTGTTATTGGG	TTGTGTTCGTGGAGGCTTTT
MyHC	GCACGGCAACTGCTACAACA	CAGGGCGATCTGCACGAA
miR-34c mimics	AGGCAGUGUAGUUAGCUGAUUGC	
miR-34c inhibitor	GCAAUCAGCUAACUACACUGCCU	
ChIP assay	GACTTAAGTTGTCTCTGT	GAAACCAAGAAGAGTGTAG
Notch1 3'UTR	CTGAGTGTGATAGTGGCGTTGA	CTCTCGGCAGACCTTGAAATAG
miR-34c upstream	CTCTAATAACCGCACAATCTCG	TGTAATGTTGGCTGTCAGTCGT

Table S2. Details of the antibodies used in this study.

Primary antibody	Clone	Company	Catalog No.	Dilution
CCNB	Monoclonal	abcam	ab212977	1:2000
CCND	Polyclonal	Bioss	bs-0623R	1:700
CCNE	Polyclonal	abcam	ab33911	1:2000
N1ICD	Monoclonal	CST	# 3608	1:3000
MyoD	Monoclonal	abcam	ab16148	1:2000
Myogenin	Monoclonal	abcam	ab1835	1:2000
Myosin	Monoclonal	CST	#8824	1:3000
β-Actin	Monoclonal	Bioss	bsm-33036M	1:1000

Secondary antibody	Conjugate Used	Company	Catalog No.	Dilution
Goat Anti-mouse IgG	HRP	Bioss	bs-0296G	1:3000
Goat Anti-rabbit IgG	HRP	Bioss	bs-0295G	1:3000
